# Changes in Cooking and Breadmaking Properties of IR 841 Paddy Rice During Storage in West Africa

**DOI:** 10.3390/foods15020405

**Published:** 2026-01-22

**Authors:** Muqsita Daouda, Yann E. Madode, Santiago Arufe, Christian Mestres, Jordane Jasniewski

**Affiliations:** 1 Laboratoire de Sciences et Technologies Des Aliments, Université d’Abomey-Calavi, Abomey-Calavi 01 BP 526, Benin; 2 Université de Lorraine, LIBio, 54000 Nancy, France; 3 CIRAD, UMR Qualisud, 34398 Montpellier, France; 4 Qualisud, University of Montpellier, CIRAD, Institut Agro, IRD, 34398 Montpellier, France

**Keywords:** *Oryza sativa*, temperature, relative humidity storage duration, pasting properties, cooking ability, breadmaking ability

## Abstract

Temperature and relative humidity can significantly affect quality of paddy rice during storage. Limited studies established the link between storage time, environmental fluctuations, changes in grain and flour physicochemical properties, and culinary performances. In a West African context, IR 841 paddy rice variety was stored under humid–sub-humid (HSH), and dry (DRY) conditions for 12 months. Over 12 months, rice stored under DRY conditions experienced greater environmental fluctuations than rice stored under HSH conditions. Grain water absorption capacity (WAC) increased during storage under DRY conditions, rising from 3.3 ± 0.3 to 3.8 ± 0.3 g/g DM between 0 and 12 months. Flour amylose content and soluble solids remained relatively stable from month 0 to 6 in all conditions, and further under HSH conditions. The observed changes led to improved grain cooking performance after 6 months of storage under DRY conditions. After 12 months, a decrease in rice flour WAC and a peak in viscosity were observed, while mean particle size increased from 42 ± 1 to 67 ± 3 μm under HSH conditions and from 31 ± 3 to 83 ± 3 μm under DRY conditions. Storage time may reduce the breadmaking capacity of rice flour. Overall, environmental fluctuations under DRY conditions strongly affected rice grain and flour properties.

## 1. Introduction

Rice production and consumption are increasing rapidly worldwide. Over the last decade, global production has increased to 800 million metric tons in 2023 [1]. A similar trend was noticed in West Africa, where local production significantly grew from 16 to 24 million metric tons from 2013 to 2023 [1]. The West African demand for rice is estimated at 38 million metric tons in 2023 [2]. Therefore, to fill the gap between production and demand, most West African countries currently import rice and promote local rice production and use. In Benin, “IR 841” is the most preferred variety due to its high yielding, long and white grain, naturally perfumed properties [3]. Houngbédji and colleagues (2018) [4] measured its amylose content as low (18.3 ± 0.4%). The variety is generally used for processing into parboiled and/or milled rice and is stored year-round either as paddy or milled grain.

In West Africa, paddy rice is generally stored loose in granaries or bagged in warehouses in sewn polyethylene bags (used by almost all Beninese farmers) or jute bags stored on wooden pallets [5]. In Benin, most farmers (73.8%) store rice paddy in their households, while 26.2% store it in warehouses, primarily using polyethylene bags [6]. Apart from packaging, environmental conditions such as temperature, relative humidity and gas composition as well as storage duration can highly influence rice quality. Humidity level and storage place promote the growth of biological agents (insects, mould, etc.) and formation of undesirable chemical compounds (mycotoxin) [5]. Moreover, quantitative and qualitative damage such as broken grain, weight loss, loss of nutritional value, foul odour, discoloration, increase in moisture content, low germination rates, and lipid oxidation can be observed [5,7,8]. Physical and functional properties, and further cooking and eating quality, of rice grain and flour can also be affected. Amylose content is the most important single index for predicting rice cooking and processing performance [8]. Gao and colleagues (2024) [9] noticed a positive correlation between amylose content and cooked rice hardness. Araki and colleagues (2016) [10] observed that low amylose content rice (6–10%) produced soft bread, with a chewy texture, while intermediate amylose content (16–20%) produced chewy bread with moderate softness, better shape and specific volume. Amylose content can vary during storage. Increase in amylose content was noticed in low intermediate and high amylose content rice after 3 months of storage at 20 °C [8]. By contrast, in the study of Gu and colleagues (2019) [11] on low amylose content (18%) rice during one year at 25 °C, a decrease in amylose content from 18 to 13% was observed. Tian and colleagues (2025) [12] showed a high physicochemical stability of low-amylose (7–11%) rice varieties during paddy storage after one year of storage at a daily temperature varying between 7 and 39 °C. The rice flour storage of low amylose content (17%) at room temperature (20 °C) for 3 months also showed no quality alteration [8]. For high and low amylose content varieties, rice milling yield was generally stable during storage whereas rice appearance quality (including chalkiness, appearance value) decreased [12]. During paddy rice storage for 12 months at 30 ± 2 °C and 80 ± 5% of relative humidity (RH), an increase in moisture content, head rice, polishing yield, amylose content and free fatty acid in rice grains as well as a darkened colour were noticed [13]. Under the same conditions for 9 [14] and 12 months [13], rice grain water uptake ratio and hardness increased while grain soluble solid losses decreased, resulting in improved cooking properties. Less variation was noticed for gelatinization temperature, which remained stable during one year of low amylose rice storage at 25 and 37 °C [14]. In regard to pasting properties, more variations were observed depending on storage conditions. Zhu and colleagues (2023) [15] noticed a decrease in peak viscosity while an increase was found by Shu and colleagues (2021) [14] after one year of storage at a high temperature (>30 °C). In the study of Prasantha and colleagues (2014) [13], pasting temperature decrease while it was stable in the research of Zhu and colleagues (2023) [16] and increased in the work of Shu and colleagues (2021) [14] after one year of storage at a high temperature. Prasantha and colleagues (2014) [13] observed an increase in breakdown whereas breakdown and setback decreased, which can vary rice flour and grain cooking ability. Rice appearance parameters are determinant of its cooking and eating quality. Rice with high milling yield, high head rice, whiteness, low chalkiness and high appearance quality is generally preferred by West African consumers [17]. Rice particle size between (106–180 μm) also shows a significant effect on bread properties, with a higher bread specific volume and lowest hardness [18]. It indicated that starch granules were damaged, thus leading to declined integrity with the extension of storage time scanning electron microscopy (SEM) [19].

In West Africa, limited data is available on paddy rice physico-chemical properties and culinary behaviour during long-term storage under local environmental conditions. Houssou and colleagues (2024) [20] reported after a six-month storage study in southern Benin (humid–sub-humid conditions) limited changes in moisture content, ash and protein contents, water activity, water absorption capacity, alkaline digestibility, and sensory aroma of native and parboiled IR 841 paddy rice. The influence of storage conditions (temperature and relative humidity) on the physicochemical properties, cooking and breadmaking ability of locally grown West African rice varieties have been poorly investigated. Such data could contribute to their better utilization.

This study examines variations in temperature and relative humidity in warehouses under contrasted conditions (dry and humid–sub-humid climates) in Benin over 12 months of paddy rice storage, and their impact on the technological suitability of rice grain and rice flour. These data are expected to help define a minimum storage time before processing under West African climate conditions, benefiting consumers, the rice industry and enhancing the value of locally grown rice.

## 2. Materials and Methods

### 2.1. Experimental Design

Rice variety IR 841 was used as the plant material for this study. Paddy rice was stored in polyethylene bags in two contrasting climatic zones of Benin. The first was in humid–sub-humid conditions (HSH) characterized by variations in temperature and relative humidity of 24 to 30 °C and 70 to 90%, respectively, during the day [21]. The second was in dry conditions (DRY) characterized by variations in temperature and relative humidity of 26 to 34.3 °C and 26 to 82% respectively, during the day [22]. As shown in Supplementary Material Table S1, three (3) warehouses with similar characteristics were used per climatic condition as biological replicates. In HSH conditions, the geographic coordinates of the three warehouses were 7.17037° N, 2.54008° E; 6.56705° N, 2.55472° E; and 6.27879° N, 1.80458° E. In DRY conditions they were 11.80350° N, 3.38239° E; 11.79591° N, 3.38698° E; and 11.85370° N, 3.29398° E. A total of 50 kg of paddy rice freshly harvested after cultivation in the same climatic zone (12–14 g/100 g moisture content) was stored in polyethylene bags on wooden pallets. For 12 months (February 2023 to February 2024), a monthly sampling of 2 kg was conducted during the first 6 months, named T0, T1, T2, T3, T4, T5, and T6, and quarterly sampling was conducted over the last 6 months, called T9 and T12 at the upper, central, and lower sections of each bag. Sampling was performed monthly during the first six months of storage to capture rapid early changes, and quarterly during the last six months, when variations were slower and more stable based on previous studies of Baoua and colleagues (2016) [23] and Chitsuthipakorn and colleagues (2023) [24]. Each batch of 2 kg of paddy rice sample was dehulled three times for 5 min with a total duration of 15 min, using a Satake husker (Satake Corporation, Hiroshima-ken, Japan). Then, it was polished once for approximately 20 min using a Yamamoto Testing Whitener VP-32T (Yamamoto Co., Ltd., Yamagata, Japan) at a whiteness level of 4.5 and a flow rate of 2. During milling, the husking yield (weight in grams of cargo rice obtained from 100 g of paddy rice) and polished yield (weight in grams of white rice obtained from 100 g of paddy rice) were calculated. Rice flour was produced for physicochemical analysis using a Retsch ZM 200 grinder (Haan, Germany), equipped with an 80 μm sieve and set at 12,000 rpm for 1 min.

### 2.2. Monitoring of Storage Conditions

An I-button electronic easy log (EL-USB-2 of Lascar Electronics, Salisbury, UK) memory stick was inserted in the middle of one bag per warehouse, and a second one was placed in each warehouse at a distance of 2 m from the rooftop. Temperature and relative humidity data were saved each hour. The first one measured temperature (°C) and relative humidity (%) inside the paddy rice bags, and the second measured the same parameters in the warehouse during storage for 3 to 12 months (May 2023–February 2024) due to logistic delays in I-button delivery, while rice was already harvested for storage.

### 2.3. Physical Properties of Stored Paddy Rice

#### 2.3.1. Head Rice, Moisture Content, and Colour

Head rice (the whole grains of white rice that can be obtained from a given quantity of clean paddy or broken rice larger than 3/4 of a grain) was determined by sieving 25 g of white rice 5 times after milling. Then, broken rice was separated into head rice, and the percentage of head rice was calculated in triplicate using Equation (1).

(1) $$\mathrm{Head}\;\mathrm{rice}\;(\%)=\frac{\mathrm{Weight}\;\mathrm{of}\;\mathrm{head}\;\mathrm{rice}}{\mathrm{Initial}\;\mathrm{weight}\;\mathrm{of}\;\mathrm{white}\;\mathrm{rice}}\times 100$$

Moisture content of rice flour was determined in triplicate using the method of Latimer (2023) [25] based on the difference between the weights before and after drying the samples at 105 °C for 72 h. The colour of white rice was measured using chromatic coordinates L* (lightness), a* (redness), and b* (yellowness), as well as delta E (ΔE*: colour difference) concerning a white ceramic standard (Y = 86.10; x = 0.3194; y = 0.3369) using a Konica Minolta CR 410 chromameter, Japan. The three measures were realized in triplicate.

#### 2.3.2. Scanning Electron Microscopy of Rice Flour

Scanning electron microscopy (SEM) Hitachi S-4800 (Hitachi, Ltd., Tokyo, Japan), optimized for high resolution, was used to visualize the structure and size of rice flour sample particles. One gram of flour was metallized with carbon 48 h before analyzing. The acceleration voltage was set at 3–5 kilovolts, magnification at 400–15,000, and resolution at 6–8 mm. Particles were captured at a scale of 10–100 μm, either individually or as agglomerates, and their size was measured using ImageJ software (version 1.54 java 13.0.6) with a 1 μm resolution of 256 pixels per unit.

#### 2.3.3. Rice Flour Particle Size Measurement of Rice Flour

Laser granulometry has been used to determine the size of rice sample particles using the Mastersizer 3000, equipped with the Aero S dispersion system (Malvern Panalytical, Worcestershire, UK), and its associated software. A hopper gape of 2 mm, a feed rate of 62%, an air pressure of 2 bars, starch-like material, one measure per sequence, and a residual value inferior to 2% were applied. In total, 15–20 g of the rice sample was analyzed three times. D50, the mean size of the particle, was calculated, and the measurement was repeated in triplicate.

### 2.4. Functional Properties of Rice Samples

#### 2.4.1. Grain Water Absorption Capacity and Soluble Solid Losses

A total of 10 g of whole grains was cooked in 250 mL of boiling distilled water for 20 min according to the Benin National Catalogue of plant species [26]. Rice grain water absorption capacity (*WAC_g_*) and solid soluble losses (*SSL*) were determined, respectively, following the methodologies of Singh and colleagues (2005) [27] and Sidhu and colleagues (1975) [28]. Both were evaluated three times per sample and calculated with the formulas presented in Equations (2) and (3):

(2) $$WACg(\frac{g\;water}{g\;dry\;matter})\;=\;\frac{Uncooked\;rice\;weight-Cooked\;rice\;weight}{\left(Uncooked\;rice\;\times \;Cooked\;rice\right)dry\;matter}\;\times \;100$$

(3) $$SSL\;\left(\%\right)=\frac{Dried\;cooked\;rice\;weight}{Cooked\;rice\;weight}\times 100$$

#### 2.4.2. Flour Water Absorption Capacity and Water Solubility Index

The flour water absorption capacity (*WAC_f_*) and water solubility index (*WSI*) were determined based on Yousf and colleagues’ (2017) [29] method modified as follows: 1 g of flour mixed with 30 mL of distilled water was saturated for 40 min at 170 rpm in an incubator (GFL 3031), and centrifuged at 2000× *g* for 10 min using a VWR MEGA STAR 600R centrifuge (VWR International, Leuven, Belgium) both at 30 °C. The pellet and supernatant were weighed. Moisture content of the supernatant was determined after 24 h at 105 °C. Three repetitions of each measure were realized, and Equations (4) and (5) were used to calculate the two parameters.

(4) $${\mathrm{WAC}}_{f}(\mathrm{mL}/g)=\frac{30\text{-}\mathrm{Supernatant}\;\mathrm{volume}\;}{1\;}$$

(5) $$\mathrm{WSI}(\%\mathrm{MS})=\frac{\mathrm{Dry}\;\mathrm{supernatant}\;}{\mathrm{Initial}\;\mathrm{weight}\;\mathrm{of}\;\mathrm{supernatant}\;}\times 100$$

#### 2.4.3. Rheological Properties

Rheological properties were assessed in triplicate as described by Bouniol and colleagues (2021) [30] using a Haake ViscotesterTM iQ rheometer (Longwood, FL, USA). Rice flour (approximately 2 g, depending on moisture content) was suspended in 20 mL of distilled water to reach 8% dry basis suspension based on the study of Bouniol and colleagues (2021). This suspension was heated at 35 °C for 1 min, raised to 50 °C in 1 min at a speed of 14 °C/min, then raised to 95 °C in 7.5 min, then held for 5 min, and finally cooled to 35 °C in 10 min, and held for 2 min. During the entire process, the suspension was stirred at 160 rpm with a double helix geometry FL 26 2B/SS. Pasting temperature (PT: temperature at the viscosity > 20 mPa·s), peak viscosity (PV), final viscosity at 50 °C (FV), ease of cooking (time to PV-Pasting time), breakdown (PV–minimum viscosity), and setback (FV—minimum viscosity) were generated by the Haake ViscotesterTM iQ rheometer programme.

#### 2.4.4. Gelatinization Temperature

Gelatinization temperature (GT) was measured in triplicate with Differential Scanning Calorimetry (DSC 250, TA Instruments, Waters France, Guyancourt, France), following the combined methodologies of Mestres and colleagues (2011) [31] and Jung and colleagues (2017) [32]. Two milligrams of rice flour from each variety was mixed with 10 µL of distilled water in a hermetically T Zero sealed aluminum sample pan (TA Instruments). The analysis was conducted by heating from 20 °C to 100 °C at a rate of 10 °C/min, followed by rapid cooling from 100 °C to 20 °C at a rate of 100 °C/min. The enthalpy of gelatinization and the gelatinization temperature were determined using TRIOS software v5.1.146572 (TA Instruments).

#### 2.4.5. Amylose Content

The amylose content (AC) in rice flour was determined in triplicate using the method by Mestres and colleagues (2011) [31], which measures the energy required for the formation of the amylose/lysophospholipid complex by Differential Scanning Calorimetry (DSC 250, TA Instruments, Waters France). L-a-Lysophosphatidylcholine (LPC) from egg yolk (L-4129, Sigma-Aldrich, Saint-Quentin-Fallavier, France) solution (2% *w*/*v* in distilled water) was mixed with rice flour and heated from 35 °C to 160 °C at 15 °C/min, kept at this temperature for 2 min, and then decreased to 60 °C at 10 °C/min. A pure amylose was used as a standard, and the ratio of energy of the sample to that of the amylose standard is used to calculate the amylose content.

### 2.5. Statistical Analysis

The effect of storage time on the properties of stored rice grain and flour was determined by analysis of variance (one-way) in humid–sub-humid (HSH) and dry (DRY) conditions considered as fixed factors. The Tukey post hoc test (*p* < 0.05), processed using Minitab 2019, and verified for the normality of the test and heterogeneity of variance were used. Flour amylose content, flour rheological properties (pasting temperature, peak of viscosity, final viscosity, breakdown, setback, and ease of cooking), grain water absorption capacity, grain colour, and grain head rice were used to realize a multiple linear regression model with storage bag temperature and relative humidity in the two conditions based on the studies of Mané and colleagues (2021) [17], Mestres and colleagues (2011) [31], Bleoussi and colleagues (2016) [33], and Dahdouh and colleagues (2021) [34]. Physicochemical parameters were considered as dependent variables, while temperature and relative humidity in the storage bag were treated as independent variables in the software XLSTAT 2019.

## 3. Results

### 3.1. Temperature and Relative Humidity Variation During Paddy Rice Storage

**Figure 1:**
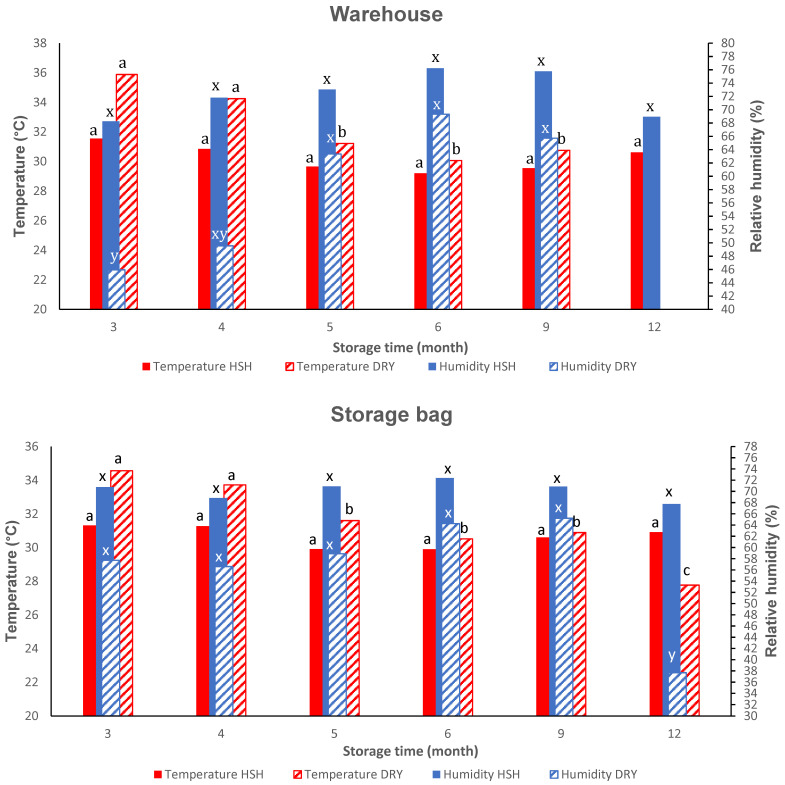
Changes in mean daily temperature and relative humidity inside the warehouse and storage bag occur under HSH and DRY conditions at T3, T4, T5, T6, T9, and T12 storage months. Legend: Data value of each bar with different superscript letters per colour is significantly different, *p* ≥ 0.05; DRY: dry conditions; HSH: humid–sub-humid conditions.

**Figure 2:**
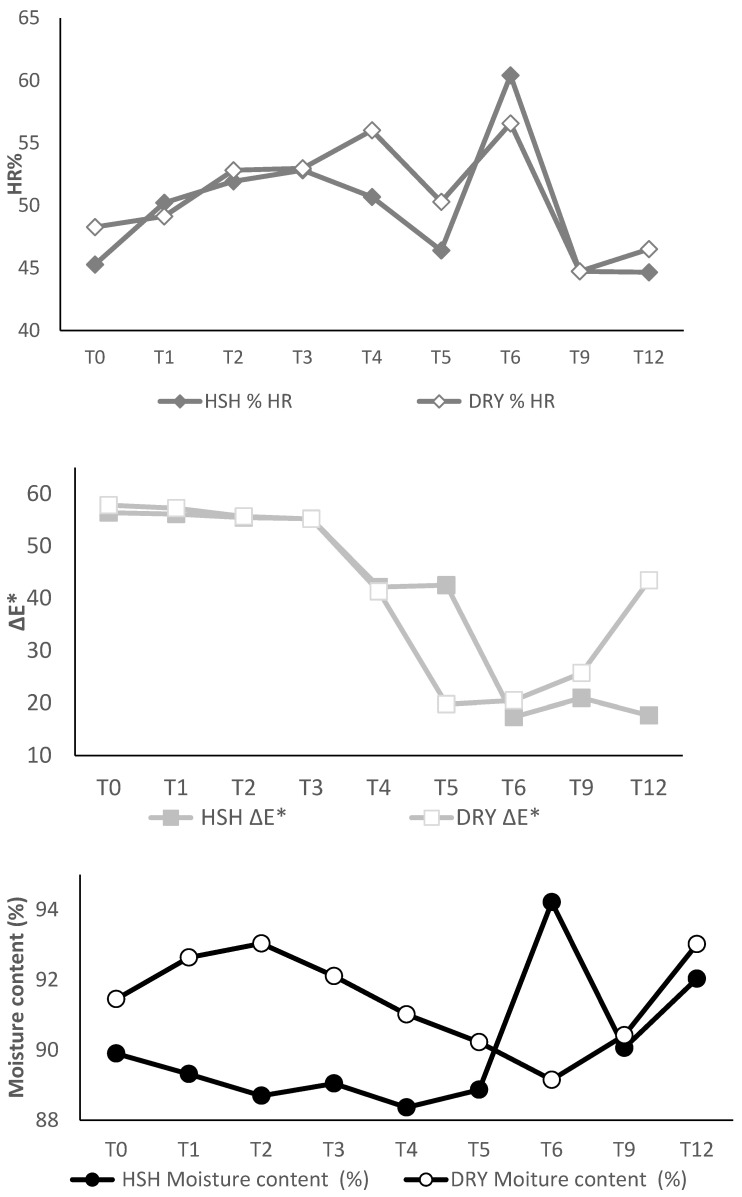
Changes in head rice (HR), ΔE* (colour difference with white reference), and moisture content at T0, T1, T2, T3, T4, T5, T6, T9, and T12 storage months in the HSH (humid–sub-humid) conditions and the dry (DRY) conditions.

Among environmental parameters, storage temperature and relative humidity are the most influential factors affecting the stability of grain quality. Of these two factors, relative humidity typically has a greater influence on the longevity of seeds in storage [35]. During paddy rice storage, it was observed that temperature decreased and relative humidity increased under humid–humid (HSH) and dry (DRY) conditions during 3 to 12 months of the experiment (Figure 1). Temperature and relative humidity are inversely proportional. During storage, paddy rice stored in polyethylene bags for this study was not isolated from ambient conditions. There was no significant difference in temperature and relative humidity variations within warehouses and inside the bags stored in these warehouses, under an HSH zone. Under HSH, the parameters averaged during the 9 months of data collection were 30.2 ± 0.9 °C and 30.6 ± 0.6 °C (*p* = 0.384) for temperature, and 72.3 ± 3.3% and 70.2 ± 1.6% (*p* = 0.199) for relative humidity, respectively, in the warehouse and in bags. Likewise, results were observed under dry conditions (DRY), with average temperatures of 32.4 ± 2.5 °C and 31.5 ± 2.4 °C (*p* = 0.901) and relative humidity levels of 58.8 ± 10.3% and 56.7 ± 9.9% (*p* = 0.773) for the warehouse and the storage bags, respectively. Storage time did not significantly influence temperature and relative humidity in HSH (*r*^2^ = 0.39; *p* = 0.403) and (*r*^2^ = 0.152; *p* = 0.886), respectively. More variation was noted in DRY, including an increase in relative humidity and a decrease in temperature, both in the warehouse and in the storage bag. Figure 2 presents the changes in moisture content during storage in the two conditions. There was no variation before 6 months of storage in both conditions; nevertheless, at 6 months of storage in HSH conditions, a significant increase in moisture content occurred, from 88.9% to 94.2%. The highest value of moisture content was recorded at 6 months of storage, along with the highest value of relative humidity (72.4%) and the lowest value of temperature (29.9 °C). A high relative humidity percentage was likely to decrease grain moisture content due to the osmosis phenomenon, which regulated water movement between the grain and the storage environment. By contrast, at 6 months of storage, the lowest value of moisture content was found in dry conditions, with a value of 89.1%, a high value of relative humidity (64.3%), and a low value of temperature (30.5 °C). The highest moisture content (93%) was observed at 12 months of storage, accompanied by the lowest values of relative humidity (37.7%) and temperature (27.8 °C).

### 3.2. Physical Properties After Storage Under Dry Versus Humid Conditions

Table 1 presents the parameters of milling yield and gelatinization temperature. Husking yield and gelatinization temperature (as observed in Figure S1) remained stable under both dry (DRY) and humid–sub-humid (HSH) climatic conditions. Despite significant changes in temperature during storage in HSH conditions, there was no clear trend in gelatinization. Polishing yield was maintained during storage under HSH conditions. In contrast, it was significantly but slightly reduced after 6 months of storage under DRY conditions, probably linked with the lowest grain moisture content (89.1%) (Figure 1 and Figure 2). An increase in relative humidity was observed, which could be attributed to the absorption of water by the paddy grain, resulting in a decrease in the polishing yield of rice grains.

### 3.3. Changes in Physicochemical and Functional Properties of Rice Grain During Storage Under Dry Versus Humid Conditions

#### 3.3.1. White Grain Head Rice, and Colour

Head rice (HR), colour, and moisture absorption can affect the consumer acceptability and market value of rice [17]. Changes in HR and ΔE* (colour difference with white reference) of white rice grain and flour moisture content during storage under humid–sub-humid (HSH) and dry (DRY) conditions are illustrated in Figure 2. Overall, HR varied only slightly during storage and showed similar trends under both storage conditions. However, a noticeable increase in HR was observed after 6 months of storage, reaching 60.4% and 56.6% in HSH and DRY conditions, respectively, compared to initial values of 45.2% and 48.3%. By contrast, moisture content and ΔE* showed opposite trends across storage conditions, with greater variation in ΔE*. From 0 to 12 months of storage, ΔE* decreases from 56.4 to 17.6 and from 57.8 to 43.4 in HSH and DRY conditions, respectively. ΔE* presented similar trends in both conditions; however, at 12 months, a decrease was observed in HSH, while an increase was observed in DRY. More limited changes were noticed in the moisture content, which remained stable at 0 and 12 months of storage. Nevertheless, a significant increase in HSH was observed, accompanied by a decrease in DRY, at 6 months of storage (Figure 2). The highest HR values (60.4% in HSH and 56.6% in DRY) were observed at 6 months of storage, coinciding with the lowest moisture content in DRY conditions (89.1%), whereas in HSH conditions, the highest HR corresponded to the highest moisture content (94%). From 5 months of storage to 12 months, divergent variation was observed between HSH and DRY conditions for the two parameters (Figure 2).

#### 3.3.2. Water Absorption Capacity and Solid Soluble Losses of Rice Grain and Flour

During cooking in excess water, the water absorption capacity (WAC_g_) and solid soluble losses (SSL) of white rice grains determine cooking quality and time [36]. The evolution of grain water absorption capacity and solid soluble losses of white rice as affected by 12 storage months is presented in Table 2. There was no significant difference at 0 and 12 months storage under humid–sub-humid conditions (HSH), with values of 3.3 g/g and 0.23% on a dry basis for WACg and SSL, respectively. In dry conditions (DRY), with low relative humidity (56.7 ± 9.9%), an increase in WACg from 3.1 to 3.8 g/g and a decrease in SSL from 0.24 to 0.21% of moisture content were observed. Then, storage in DRY conditions, especially after 6 to 12 months (Table 2), could improve rice grain quality as it lowers the SSL and probably cooked rice adhesiveness. The water absorption capacity (WACf) and water solubility index (WSI) of rice flour indicate its ability to bind and dissolve in water, which is generally used to predict flour suitability for breadmaking. Table 2 shows that WAC_f_ and WSI of flour decreased during the 12 months of storage in HSH and DRY conditions. Flour WACf decreased by 34% from 3.7 to 2.3 and from 3.1 to 2.1 mL/g in HSH and DRY, respectively. Flour WSI decreased by half from 0.33 to 0.15 and from 0.22 to 0.14 g/100 g moisture content basis, respectively. However, there was more variation in these parameters during storage in DRY compared to HSH, as indicated by the highest variation in temperature and relative humidity in the storage bag (Figure 1).

#### 3.3.3. Flour Granule Size (D50) and SEM

**Figure 3:**
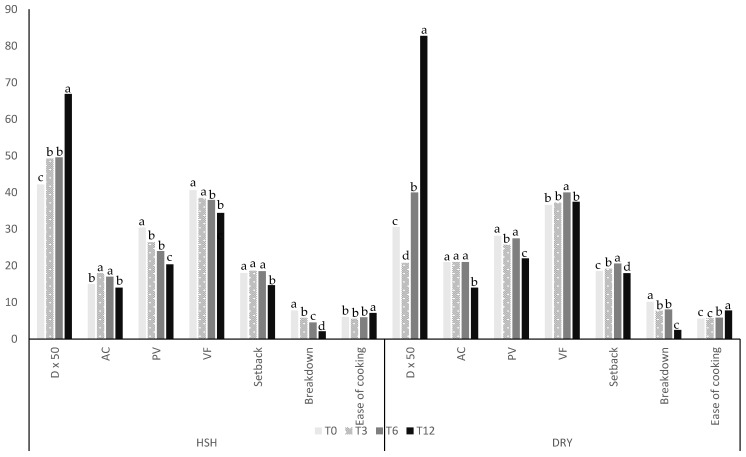
Changes in functional properties of rice flour during storage at T0, T3, T6, and T12 storage months. Legend. D50: mean of granulometry (μm); AC: amylose content (%); PV: peak of viscosity (Pa·s); VF: final viscosity (Pa·s); setback (Pa·s); breakdown (Pa·s); ease of cooking (min). The data values for each parameter with different superscript letters are significantly different.

**Figure 4:**
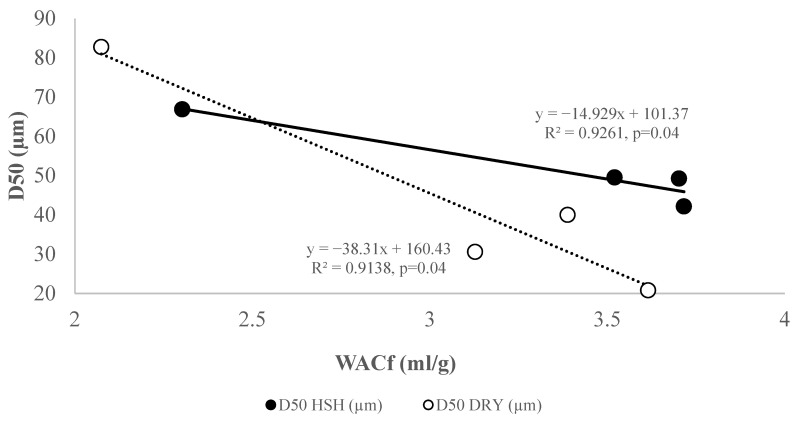
Relationship between flour particle size and water absorption capacity. Legend: WAC_f_, water absorption capacity of rice flour; HSH, humid–sub-humid conditions in South Benin; DRY, dry conditions in North Benin.

**Figure 5:**
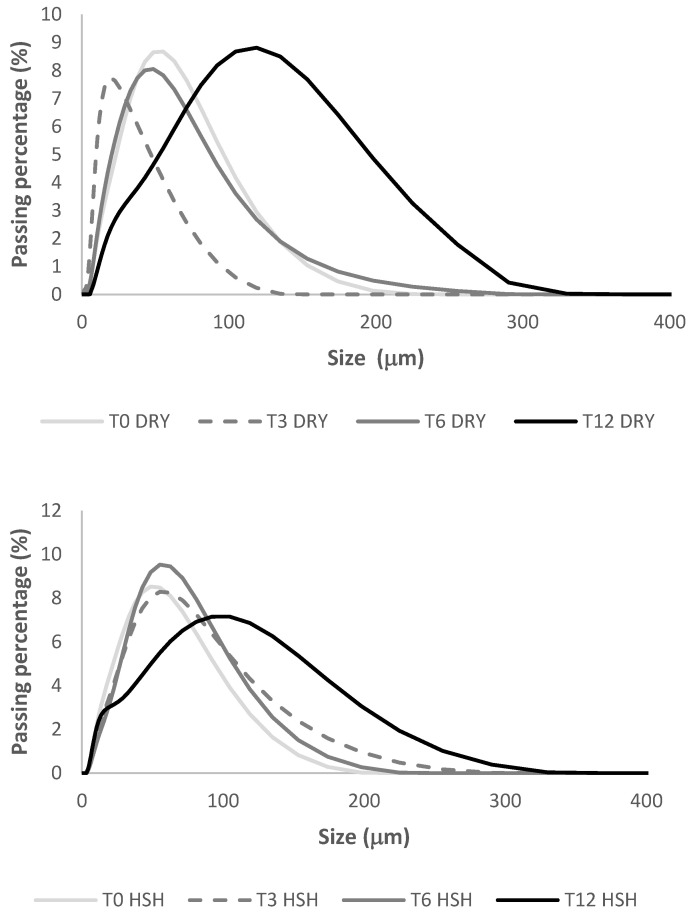
Granule size of rice stored during storage in the humid–sub-humid conditions (HSH) and the dry conditions (DRY).

D50, the mean granule particle size of rice flour (50%), was collected during storage paddy, in the humid–sub-humid conditions (HSH) and the dry conditions (DRY) and presented in Figure 3. A significant increase in D50 was observed from 0 to 12 months of storage under HSH (42 to 67 μm; 60%) or DRY (20 to 82 μm; 170%) conditions. The increase in D50 during storage affected flour water absorption capacity (WAC_f_). It was also observed that water absorption was greatest in the least dispersed particles. Flours with a lower particle size (D50) exhibited higher flour–water absorption capacities (WAC_f_) (Figure 4) for rice stored under DRY or HSH conditions. Changes in flour particle size can also be linked to variations in rheological parameters, especially the peak viscosity (PV). D50 was confirmed with granulometry curves (Figure 5) of flour over storage time. A rise in flour particle size was observed with increasing storage time, as reported in the study.

**Figure 6:**
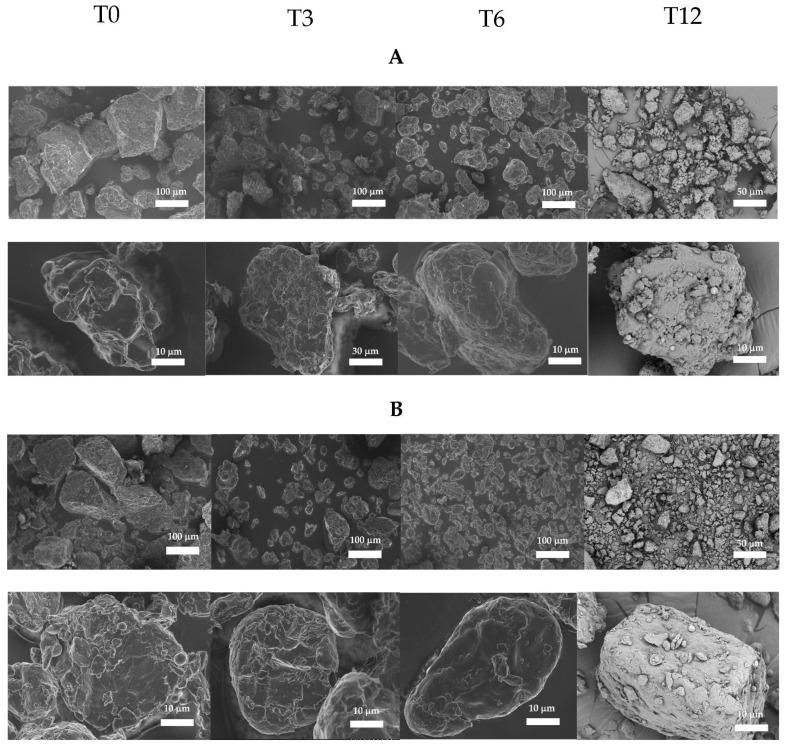
Granules’ form and distribution of rice flours by SEM as influenced by paddy storage conditions and storage time. Legend: (**A**) humid–sub-humid conditions (HSH); (**B**) dry conditions (DRY); T0, initial time; T3, 3 months of storage; T6, 6 months of storage; T12, 12 months of storage.

Figure 6 presents a scanning electron microscope (SEM) image of the distribution and granules of rice flour stored in the humid–sub-humid conditions and dry conditions at T0, T3, T6, and T12. A fragmentation or break of the particle was observed after 3 months of storage with a decrease in granule size from 3.9 to 3.5 and 3.9 to 3.1 μm in HSH and DRY conditions, respectively. Between 3 and 6 months, the granule size increased as an agglomerate formed. At 6 months, granule sizes were 4 and 4.8 μm under HSH and DRY conditions, respectively, indicating structural reorganization of flour granules. A decrease in granule size was, however, observed at 12 months of storage, with values of 3.4 and 2.7 μm in the HSH and DRY conditions, respectively. This observation contrasts with the results of D50 granulometry, which show that granule size increases with storage time. The agglomerate granules formed during 3 to 12 months of storage (Figure 6) may explain the increase in starch breakdown and granulometric size at 12 months of storage (Figure 5), despite the observed granule fragmentation. It was observed that, despite particle breakage at 3, 6, and 12 months of storage, new particles formed through granule association, and the amount of cell wall remnants gradually increased with storage at 37 °C (*p* < 0.05).

#### 3.3.4. Amylose Content and Rheological Properties

Amylose content (AC) is one of the most critical parameters in the technological aptitude of rice. After 12 months, in dry conditions (DRY), AC was stable for the first 6 months (21%) before experiencing a significant decrease at 12 months of storage (14%) (Figure S2), while it appeared not to be affected by storage time (15 to 14%) under humid–sub-humid conditions (HSH) (Figure S3). Rheological properties are generally the most affected parameters with storage conditions (Figures S4 and S5). Nevertheless, the pasting temperature remained relatively stable from 0 to 12 months of storage in humid–sub-humid (HSH) and dry (DRY) conditions. A decrease from 0 to 6 months of storage with the values of 64.6 to 62.7 °C and 62.5 to 59.8 °C, followed by an increase at 12 months of storage with the values of 64.4 and 64.5 °C in HSH and DRY, respectively, was noticed (Table 2). These findings could be linked to the decrease in flour water absorption capacity (WACf). The highest value of pasting temperature and the lowest value of WACf were observed at 12 months of storage. However, there was no significant correlation between both properties (*r*^2^ = 0.03; *p* = 0.24). A low pasting temperature results in a shorter cooking time, which is a key consideration for consumers.

Other rheological properties, such as ease of cooking, breakdown, setback, final viscosity, and peak of viscosity, measured at 0, 3, 6, and 12 months of storage under HSH and DRY conditions, are presented in Figure 3. Peak of viscosity, final viscosity, and breakdown decreased after 12 months of storage under HSH and DRY conditions. The reduction in peak viscosity value observed after 12 months (from 30.4 to 20.4 Pa·s under HSH conditions and from 28.2 to 22 Pa·s under DRY conditions of storage). Setback or starch retrogradation index remained stable for the first 6 months under HSH conditions.

## 4. Discussion

### 4.1. Storage Effect of Paddy on White Rice Cooking and Eating Capacity

Despite significant changes in temperature during storage in humid–sub-humid (HSH) conditions, there was no clear trend in gelatinization, as reported by Garofalo and colleagues (2024) [37] and Zhou and colleagues (2010) [38] after 12 months of paddy rice storage at 37 °C on three varieties. These results were also consistent with those of Prasantha and colleagues (2014) [13] showing no change in polishing yield and gelatinization temperature after 9 months of paddy rice storage at 25 °C. The stability of gelatinization temperature during storage will certainly induce a stable rice grain cooking time that the consumer is aiming at. However, there was no correlation between gelatinization temperature and cooked rice texture in previous studies. A positive, high, and significant correlation between cooked rice hardness and flour pasting temperature was noticed by Zhu and colleagues (2023) [15]; this can induce a variation in cooked rice texture during storage as pasting temperature changes are observed. The lowest values of pasting temperature were noticed at 6 months of storage, confirming previous observations on the potential increase in cooking suitability for West African consumers. However, the significantly higher pasting temperature at 12 months of storage under DRY or HSH conditions may affect rice cooking suitability. Given that these temperatures are approximately 40 °C below the boiling point of water, the impact on cookability is expected to be relatively low. The increase in relative humidity under dry (DRY) storage conditions could be attributed to water absorption by paddy grains, resulting in a decrease in moisture content and polishing yield, especially at 6 months of storage (Table 1, Figure 2). According to the study of Müller and colleagues (2022) [39], in a storage bag environment, which implies the phenomenon of osmosis through the diffusion of water from the grain to equilibrate the temperature between the grain and the environment, the rise in HR could explain the reduction in moisture content due to air–water absorption. An optimal interval of moisture content proportion should be considered before milling. In this regard, Müller and colleagues’ (2022) [39] study showed that moisture content between 77 and 82% facilitated the post-harvest stages, and high moisture content reduced milling yield. In contrast to these findings, Jang and colleagues (2009) [40] noticed an increase in paddy rice husking yield at 25 °C for 12 months, from 77% to 83%, a desirable change primarily for rice cooking quality. A decrease in ΔE* from 0 to 12 months of storage shows more stability of grain colour, potentially due to oxidation reactions or enzymatic browning. Opposite results were observed in the studies of Shafiekhani and colleagues (2018) [41] and Sung and colleagues (2014) [42] on paddy rice during storage in polyethylene bags for 4 months at temperatures from 0 to 40 °C, with a decrease in whiteness. As noticed by Nassem and colleagues (2013) [43], West African consumers preferred white rice for cooking. Colour changes noticed in this study can then enhance the acceptance of white rice among consumers in this area. The increase in grain water absorption capacity (WAC_g_) and decrease in grain solid soluble losses (SSL) during storage under DRY conditions were previously saved by Zhou and colleagues (2007) [44] during storage at 37 °C for 16 months. Similarly, Hu and colleagues (2022) [45] observed improved rice cooking properties after 12 months of storage at 30 ± 2 °C, along with a decrease in cooked rice adhesiveness, consistent with SSL, likely due to the formation of amylo–lipid complexes. Low adhesiveness is a vital criterion for cooking and eating capacity among West African consumers, which enhances its acceptability [17]. Then, storage in DRY conditions, especially after 6 to 12 months (Table 2), could improve rice grain quality as it lowers the SSL and probably cooked rice adhesiveness. A significant increase in flour D50 can be attributed to the decrease in water absorption capacity (WACf) as Lapčíková [46] observed and as illustrated in Figure 4. Qin and colleagues (2021) [47] noticed that particle size between 75 and 100 μm can exhibit preferable characteristics of rice flour, which could be used to produce similar desirable qualities of rice bread to the wet-ground rice flour. Rice flour with larger particle size had a significantly lower volume, rougher crumb structure and harder bread texture. The increase in rice particle size, specifically at 12 months of storage (Figure 5), can favour a decrease in its breadmaking ability. The reduction in amylose content (AC) in DRY conditions was also observed by Patindol and colleagues (2005) [48] after 9 months of storage for intermediate AC rice. However, AC stayed low (below 20%) throughout storage. Therefore, low amylose content rice will remain low AC rice after 12 months of storage under DRY conditions. The decrease in amylose content in DRY could be linked to the higher variation in temperature and humidity at 12 months of storage, as well as potential enzymatic degradation of amylose due to amylase activity. This decrease observed was noticed in many studies such as the one by Zhou and colleagues (2007) [44]. In contrast, Garofalo and colleagues (2024) [37] noticed an inverse trend with an increase in low amylose content rice (<22%) after 6 months of storage at 23 °C, which was considered the combined effect of temperature and air availability. Reference [15] also noticed an increase in AC after 12 months of storage at 25 °C and 75% of relative humidity. The relationship between AC and the other parameters can be observed. Chen and colleagues (2024) [8] observed a positive correlation between setback and final viscosity and amylose content for 12 months’ storage at 20 °C, as was noticed in this study (Figure 3), especially in DRY conditions. The decrease in AC could improve rice cooking suitability, with a positive correlation between AC and cooked rice hardness [15]. In fact, soft-cooked rice is preferred by West African consumers [17]. By contrast, high-amylose rice is essential for well-risen rice flour bread [15], so rice flour suitability for breadmaking can be reduced by the decrease in amylose content noticed. Peak of viscosity, final viscosity, and breakdown decreased after 12 months of storage under HSH and DRY conditions, as also shown by Hu and colleagues (2022) [19] and Jungtheerapanich and colleagues (2017) [45] on rice stored for 9 to 18 months at 25–30 °C and 70% relative humidity. The peak of viscosity is also an essential parameter for assessing rice cooking ability. A high peak of viscosity has been reported as an indicator of good breadmaking ability, while a low peak of viscosity correlates with low cooked rice adhesion and a high hardness index [15]. The reduction in peak viscosity observed in this study could improve rice cooking quality, whereas breadmaking capability decreases. Park and colleagues (2012) [49] observed an increase in setback when breakdown decreased after 4 months of storage, associated with a reduction in cooked rice taste and overall quality. Similar results were observed in this study; however, setback was more stable for the first 6 months in HSH (18 Pa·s) in comparison to DRY conditions (from 18 to 20 Pa·s at 0 and 6 months of storage). The ease of cooking rice flour reflects the starch behaviour during gelatinization and could serve as a predictor of cooked rice texture. Low ease of cooking means cooked rice is hard, and high ease of cooking means low hardness of cooked rice [50]. As illustrated in Figure 3, this parameter remained stable during the first 6 months, then increased at 12 months of storage, which may result in a decrease in cooked rice hardness. Overall, the results for changes in rice grain properties indicate that rice cooking remained stable for 6 months of storage and increased thereafter. By contrast, rice flour’s ability for breadmaking decreases with storage duration, specifically at 12 months.

### 4.2. Changes and Modelling the Cooking and Eating Capacity of Rice as Influenced by Paddy Storage Temperature and Relative Humidity

Under HSH conditions, there was no correlation (*p* > 0.05) between the parameters flour amylose content, flour rheological properties (pasting temperature, peak of viscosity, final viscosity, breakdown, setback, and ease of cooking), grain water absorption capacity, grain colour, and grain head rice and the storage conditions (temperature and relative humidity). In contrast, DRY conditions favoured a relationship between amylose content and rheological properties, temperature, and relative humidity (Table 3). The normality test revealed that only the dependent variable, amylose content residuals, did not follow a normal distribution (*p* = 0.013). However, the Durbin–Watson coefficient was close to 2 (0.964), and the Akaike Information Criterion (AIC) was −96.1, which allowed us to validate the relationship established. Using a selection of better models, the two independent variables were the most influential and explained 98% (*r*^2^ = 0.98) of the variability of amylose content with the equation:

AC = −8.5 × 10^−2^ + 5.3 × 10^−3^ × DRY temperature + 2 × 10^−3 ^× DRY relative humidity

An increase in DRY temperature and relative humidity was associated with an increase in amylose content. The two parameters had a highly significant and positive correlation with amylose content (Table 3). Flour rheological parameters were the most affected by changes in DRY temperature and relative humidity. Among rheological parameters, changes in the peak viscosity (PV), breakdown, and ease of cooking were identified as being linked to dry storage conditions. These relationships were established as follows:

PV = 13.6 + 0.3 × DRY relative humidity

Breakdown = 3.2 + 7.9 × 10^−3^ × DRY temperature × DRY relative humidity

Ease of cooking = 14 − 0.10 × DRY temperature − 0.05 × DRY relative humidity

PV, breakdown, and ease of cooking were explained, respectively, by 95%, 90% and 98% of DRY temperature and relative humidity. The adequacy of models used for the three parameters was verified with the normal distribution of residuals (*p* > 0.05), the coefficient of Durbin–Watson close to 2, and the significance of models with *p* ≤ 0.0001 (Table 3).

PV was significantly, positively, and lowly correlated with DRY relative humidity, whereas breakdown and ease of cooking were significantly, negatively, and lowly correlated with the two variables. Water absorption capacity (WAC_g_) was also associated with DRY temperature and DRY relative humidity. Garofalo and colleagues (2024) [37] also detected that grain water absorption capacity was affected by storage temperature (*p* = 0.00). In total, 78% of WAC_g_ was explained by these two independent variables (Table 3) with a negative and significant correlation, as illustrated by the equation

WAC_g_ = 6.1 − 6.5 ×10^−2^ × DRY temperature − 1.5 × 10^−2^ × DRY relative humidity

These observations are consistent with the literature, which reports that temperature and relative humidity alter the structural organization of starch granules by affecting their swelling capacity, shear stability, and cooking behaviour [38]. The significant effects of temperature and relative humidity on ease of cooking and amylose content indicate that the cooking properties of grain and flour are influenced by these two parameters. The significant effect of the temperature × relative humidity interaction on breakdown confirms that dough stability depends on the combination of storage conditions rather than their isolated effects, as previously observed for treated starches [13]. On the other hand, the absence of a significant effect on specific parameters, such as colour difference or Setback, suggests that these properties are governed more by the intrinsic composition of the raw material than by the storage conditions in the range studied [10].

The cooking and eating qualities of rice grains mainly depend on their physicochemical and functional properties. Sattari and colleagues (2015) [51] noted that high amylose cultivars (>25%) are dry and fluffy on cooking, often becoming hard after a long cooking time and cooling. Low amylose cultivars (15–20%) are soft and sticky. Intermediate amylose (20–25%) rice is smooth but not sticky and is widely preferred by most consumers. Chen and colleagues (2024) [8] found that food-grade rice with a medium-amylose content can be used to make rice noodles, which can be stored at room temperature without compromising quality or requiring additional energy. Amylose content also had a high and positive correlation with cooked rice hardness [15]. However, amylose content does not absolutely determine the texture of cooked rice. Rheological properties such as breakdown viscosity and setback viscosity can be used to evaluate the palatability differences in cooked rice with similar characteristics. Gelatinization temperature is another essential quality predictor in determining the cooking quality of rice. The time required for cooking rice is controlled by its gelatinization temperature. Rice with a low gelatinization temperature needs less energy input during cooking than rice with a high gelatinization temperature. The cooked rice water absorption capacity was also negatively linearly correlated with its hardness [52]. The quality of rice grains in terms of eating and cooking was globally stable under HSH conditions, likely due to the stability of temperature and relative humidity. In DRY conditions, rice grain properties were stable for the first 6 months. However, after 6 to 12 months of storage, changes such as a decrease in amylose content (from intermediate to low) and an increase in cooked rice water absorption capacity could lead to a reduction in cooked rice hardness.

This study established that these changes were linked to variations in temperature and relative humidity during storage, exhibiting a negative relationship with water absorption capacity. The most critical parameters of rice flour that define the breadmaking performance for gluten-free bread were swelling capacity and gelatinization temperature. Flour properties, rheological properties such as setback and breakdown, were strongly related to the cohesiveness value of gluten-free bread. Also, high swelling power and low breakdown viscosity of the rice flour were associated with high specific volume of the gluten-free bread [53]. Another study of Han and colleagues (2012) [54] indicated that high or intermediate amylose content and low water absorption were the primary indicators of rice bread flour quality. Changes in flour’s physical and functional properties were observed with a decrease in rheological properties and water absorption capacity in HSH and DRY conditions, which potentially led to an increase in flour breadmaking performance specifically for conservation. By contrast, a decrease in amylose content could induce a reduction in breadmaking performance. However, flour properties were stable for the first 6 months of storage.

## 5. Conclusions

Changes in IR 841 paddy rice properties (cooking and breadmaking abilities) during twelve months’ storage were investigated as a factor of temperature and relative humidity. The final goal was to define a minimum paddy rice storage time before processing it under West African climate conditions, for the benefit of the local rice industry and consumers.

A greater stability in temperature, relative humidity, and in the physicochemical properties and cooking aptitude of rice grains was observed during storage under HSH conditions. Storage of paddy rice under stable conditions, with high humidity and low temperature, can preserve the technological quality of the rice grain. In contrast, under DRY conditions, variation in temperature and relative humidity appeared to increase grain water absorption capacity, and decrease amylose content, which could improve rice grain potential suitability for cooking, especially after 6 months of storage. Consequently, IR 841 offers the best cooking properties from six months onwards. Therefore, the minimum storage time no matter the storage conditions is suggested to be six months.

The properties of flour were less stable during storage under both conditions. Changes in water absorption capacity of flour, water solubility index, peak viscosity, setback, breakdown, ease of cooking, and particle size, observed in the two conditions, altered the breadmaking potential of flour, with greater changes observed at 12 months of storage. For breadmaking, it might be preferable to mill rice directly after harvesting to preserve its properties and suitability. Therefore, no storage is recommended if IR 841 is to be used for breadmaking.

## Figures and Tables

**Table 1:** Physical characteristics of rice as affected by paddy storage conditions and time.

Storage Time	HSH			DRY		
	Husking Yield (%)	Polishing Yield (%)	GT (°C)	Husking Yield (%)	Polishing Yield (%)	GT (°C)
T0	77 ± 1 ^a^	58 ± 3 ^a^	72 ± 1 ^a^	77 ± 2 ^a^	60 ± 3 ^abc^	70 ± 1 ^a^
T1	76 ± 1 ^a^	60 ± 2 ^a^		77 ± 2 ^a^	63 ± 1 ^bc^	
T2	77 ± 1 ^a^	62 ± 2 ^a^		76 ± 1 ^a^	63 ± 1 ^ab^	
T3	76 ± 2 ^a^	62 ± 2 ^a^	72 ± 1 ^a^	76 ± 2 ^a^	64 ± 1 ^a^	70 ± 1 ^a^
T4	74 ± 2 ^a^	57 ± 2 ^a^		75 ± 2 ^a^	60 ± 2 ^abc^	
T5	74 ± 4 ^a^	54 ± 4 ^a^		74 ± 4 ^a^	57 ± 4 ^abc^	
T6	74 ± 1 ^a^	56 ± 2 ^a^	71± 1 ^a^	78 ± 1 ^a^	53 ± 3 ^c^	71 ± 1 ^a^
T9	75 ± 2 ^a^	58 ± 4 ^a^		75 ± 2 ^a^	62 ± 5 ^ab^	
T12	74 ± 3 ^a^	57 ± 4 ^a^	72 ± 1 ^a^	74 ± 3 ^a^	61 ± 3 ^abc^	69 ± 1 ^a^
*p* -value	0.032	0.732	0.100	0.181	<0.001	0.091

Legend. Data are presented as mean ± standard deviation. The data value of each parameter with different superscript letters in columns is significantly different, *p* ≥ 0.05; GT: gelatinization temperature; DRY: dry conditions; HSH: humid–sub-humid conditions.

**Table 2:** Functional characteristics of rice as affected by paddy storage conditions and time.

Storage Time	HSH						DRY			
	WAC_f_ (mL/g)	WSI (g/100 g DM)	WAC_g_ (g/g DM)	SSL (% DM)	Pasting Temperature (°C)	WAC_f_ (mL/g)	WSI (g/100 g DM)	WAC_g_ (g/g DM)	SSL (% DM)	Pasting Temperature (°C)
T0	3.7 ± 0.4 ^ab^	0.33 ± 0.15 ^a^	3.2 ± 0.1 ^ab^	0.23 ± 0.01 ^c^	64.6 ± 1.6 ^ab^	3.1 ± 0.7 ^c^	0.22 ± 0.08 ^a^	3.1 ± 0.2 ^bc^	0.24 ± 0.01 ^bc^	62.5 ± 1.0 ^b^
T1	3.8 ± 0.6 ^a^	0.24 ± 0.08 ^b^	3.1 ± 0.1 ^b^	0.23 ± 0.01 ^c^		3.9 ± 0.5 ^ab^	0.27 ±0.05 ^bcd^	3.3 ± 0.3 ^b^	0.23 ± 0.02 ^c^	
T2	3.3 ± 0.6 ^bc^	0.26 ± 0.08 ^b^	2.9 ± 0.1 ^cd^	0.25 ± 0.01 ^ab^		3.6 ± 0.9 ^bc^	0.31 ± 0.06 ^bc^	3.0 ± 0.1 ^cd^	0.25 ± 0.02 ^ab^	
T3	3.7 ± 0.6 ^ab^	0.29 ± 0.05 ^b^	3.0 ± 0.1 ^c^	0.24 ± 0.01 ^b^	65.7 ± 1.2 ^a^	3.6 ± 0.3 ^bc^	0.28 ± 0.03 ^ab^	3.1 ± 0.1 ^bc^	0.23 ± 0.02 ^c^	63.2 ± 1.1 ^b^
T4	3.0 ± 0.5 ^cd^	0.21 ± 0.03 ^b^	2.9 ± 0.1 ^c^	0.25 ± 0.01 ^b^		3.1 ± 0.8 ^c^	0.21 ± 0.03 ^cd^	2.9 ± 0.2 ^cd^	0.25 ± 0.01 ^ab^	
T5	3.9 ± 0.6 ^a^	0.22 ± 0.06 ^b^	2.8 ± 0.1 ^d^	0.25 ± 0.01 ^a^		4.4 ± 0.4 ^a^	0.21 ± 0.04 ^cd^	2.9 ± 0.1 ^d^	0.25 ± 0.01 ^a^	
T6	3.5 ± 0.9 ^abc^	0.22 ± 0.02 ^bc^	2.9 ± 0.1 ^cd^	0.25 ± 0.00 ^a^	62.7 ± 0.6 ^c^	3.4 ± 0.5 ^bc^	0.20 ± 0.05 ^cd^	3.3 ± 0.3 ^b^	0.23 ± 0.02 ^c^	59.8 ± 1.1 ^c^
T12	2.3 ± 0.2 ^d^	0.15 ± 0.02 ^c^	3.3 ± 0.3 ^ab^	0.23 ± 0.01 ^c^	64.4 ± 0.2 ^b^	2.1 ± 0.1 ^d^	0.14 ± 0.01 ^d^	3.8 ± 0.3 ^a^	0.21 ± 0.02 ^d^	64.5 ± 0.1 ^a^

Legend. Data are presented as mean ± standard deviation. Data value of each parameter with different superscript letters in columns is significantly different; WAC_f_: water absorption capacity of flour; WSI: water solubility index of flour; WAC_g_: water absorption capacity of grain; SSL: solid soluble losses of grain; % DM: g/100 g of dry solid.

**Table 3:** Summary of model equation and *p*-values.

	∆E*	% HR	WACg	Pasting Temperature	PV	VF	Breakdown	Setback	Ease of Cooking	AC
R^2^	0.138	0.322	0.660	0.493	0.952	0.134	0.897	0.250	0.978	0.978
F	0.482	1.422	5.833	2.918	59.032	0.463	26.039	1.003	136.121	134.844
Pr > F	0.639	0.312	0.039	0.130	0.000	0.650	0.001	0.421	<0.0001	<0.0001
DRY temperature		1.917	4.188				3.542		55.227	55.519
		0.216	0.087				0.109		0.000	0.000
DRY relative humidity	0.937		3.684	3.379	26.588	0.891		0.293	132.455	130.208
	0.370		0.103	0.116	0.002	0.382		0.608	<0.0001	<0.0001
DRY temperature × DRY relative humidity	0.927	0.024		1.320	1.847	0.662	42.763	0.001		
	0.373	0.881		0.294	0.223	0.447	0.001	0.975		

Legend. *R*^2^, coefficient of determination; *F*, Fisher’s test statistic; *Pr*, *p*-value; ΔE*, colour difference with white reference; %HR, head rice; grain WAC_g_, water absorption capacity of rice grains; PV, peak of viscosity; VF, final viscosity; AC, amylose content.

## Data Availability

The original contributions presented in the study are included in the article/Supplementary Materials, further inquiries can be directed to the corresponding author.

## Supplementary Materials

The following supporting information can be downloaded at: https://www.mdpi.com/article/10.3390/foods15020405/s1, Figure S1: Gelatinization curves during storage; Figure S2: Amylose curves during storage in DRY conditions; Figure S3: Amylose curves during storage in HSH conditions; Figure S4: Rheological curves during storage in DRY conditions; Figure S5: Rheological curves during storage in HSH conditions; Table S1: Warehouses characteristics.
