# Supplementary material for: Changes in Cooking and Breadmaking Properties of IR 841 Paddy Rice During Storage in West Africa
Source: Foods. 2026 Jan 22;15(2):405. doi: 10.3390/foods15020405 (PMC12840959; doi:10.3390/foods15020405)
Supplement: Supplementary file 1 [file foods-15-00405-s001.zip › foods-4055192-supplementary.pdf]

Supplementary material

Table S1. Warehouses characteristics

Characteristics	Warehouse	
	**Humid sub-humid**	**Dry conditions**
**Warehouse size (m)**	Length: 21.6<br>Width: 10.8<br>Height: 8.6	Length: 22<br>Width: 11<br>Height: 10
**Topography**	Raised above ground level, bushland surroundings	
**Exposure to sunlight**	Face the sun	
**Warehouse shape**	Rectangular	
**Roof**	Shed roof	
**Building materials**	Cement	
**Warehouse opening door (m)**	4.6x4.6	3.5x3.5
**Warehouse opening window**	Perforation of the wall covered with wire mesh close to the roof	
**Pallet size (m)**	1.2 x 0.9	
**Storage bags arrangement**	Perpendicularly	
**Aeration**	1 m between stacks of bags and 1 m between bags and wall	

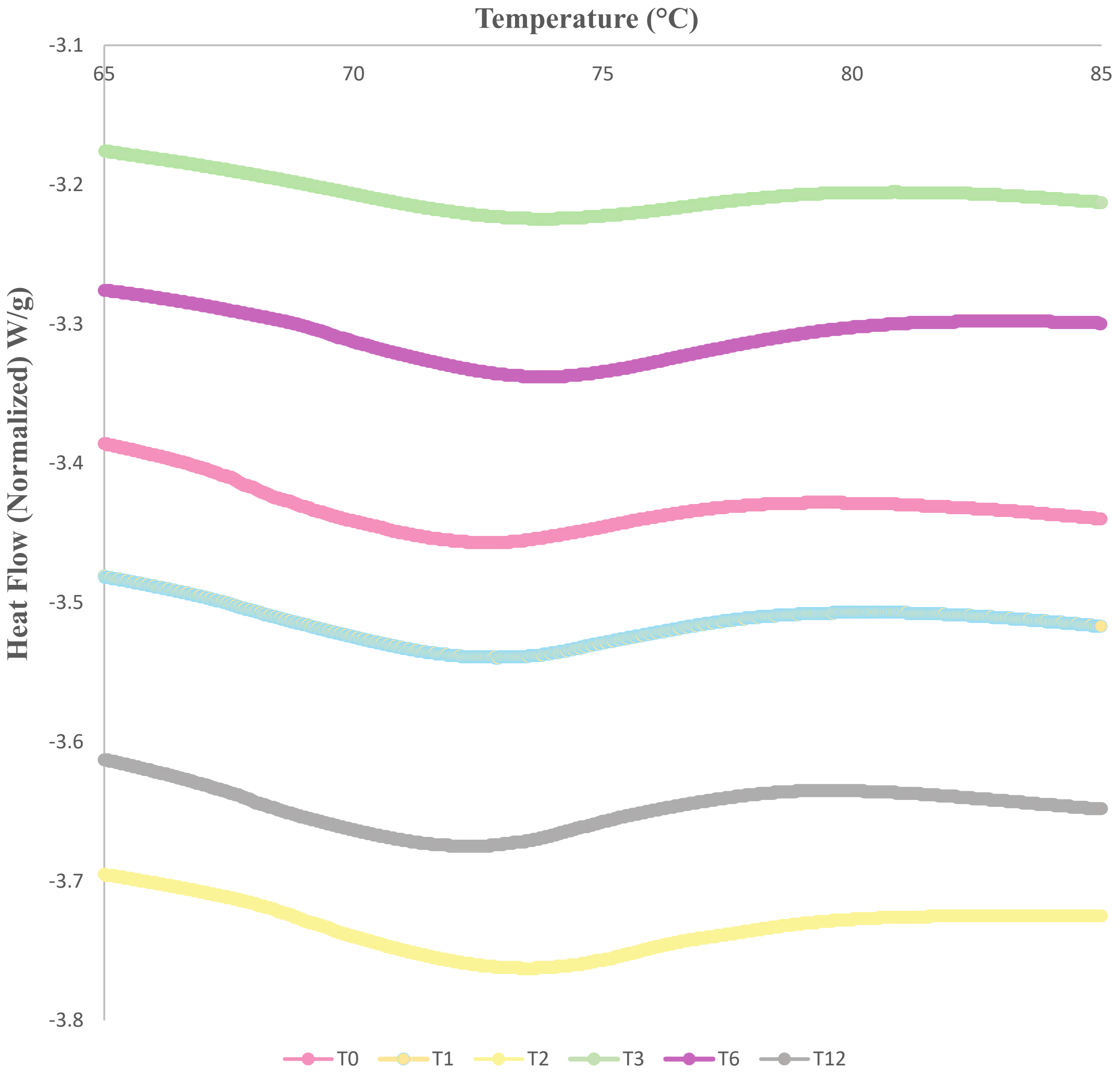

Figure S1. Gelatinization curves during storage

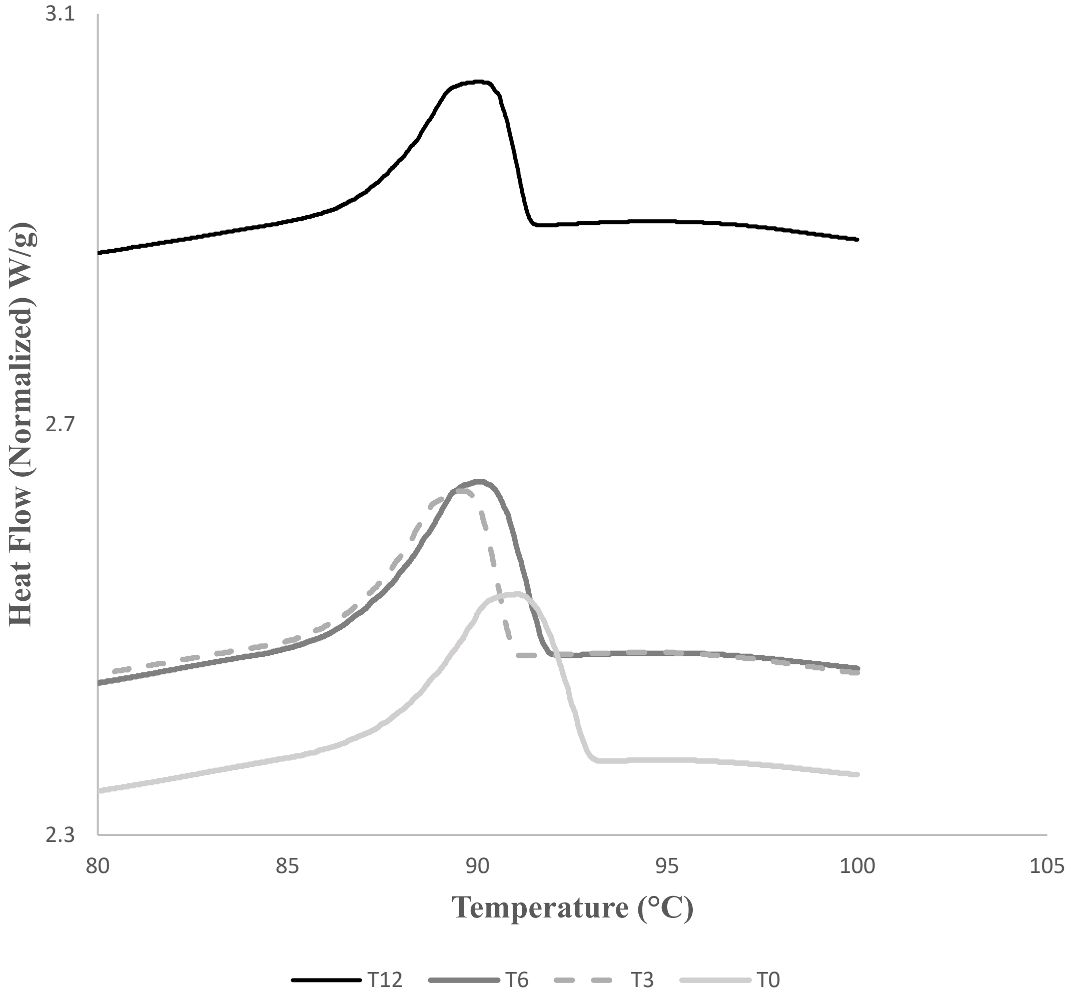

Figure S2. Amylose curves during storage in DRY conditions

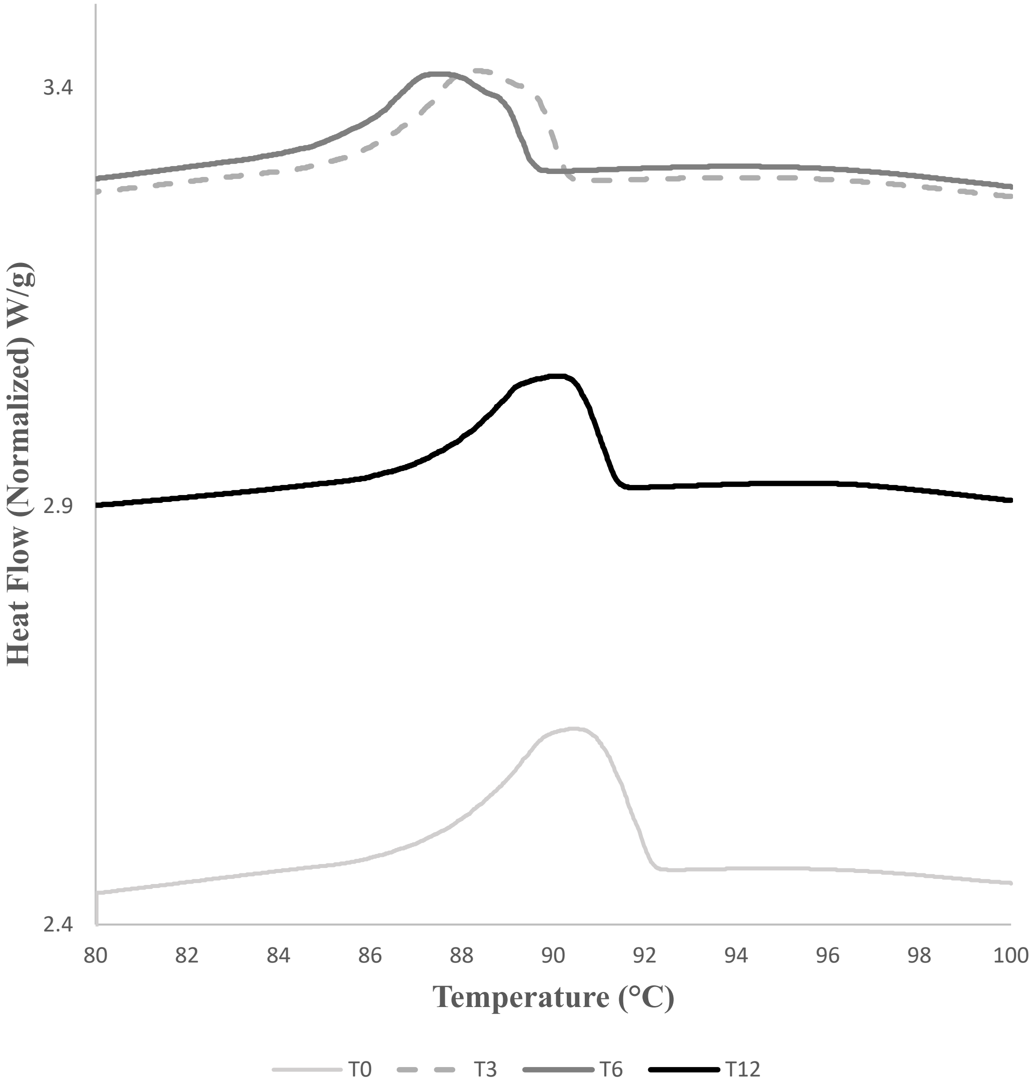

Figure S3. Amylose curves during storage in HSH conditions

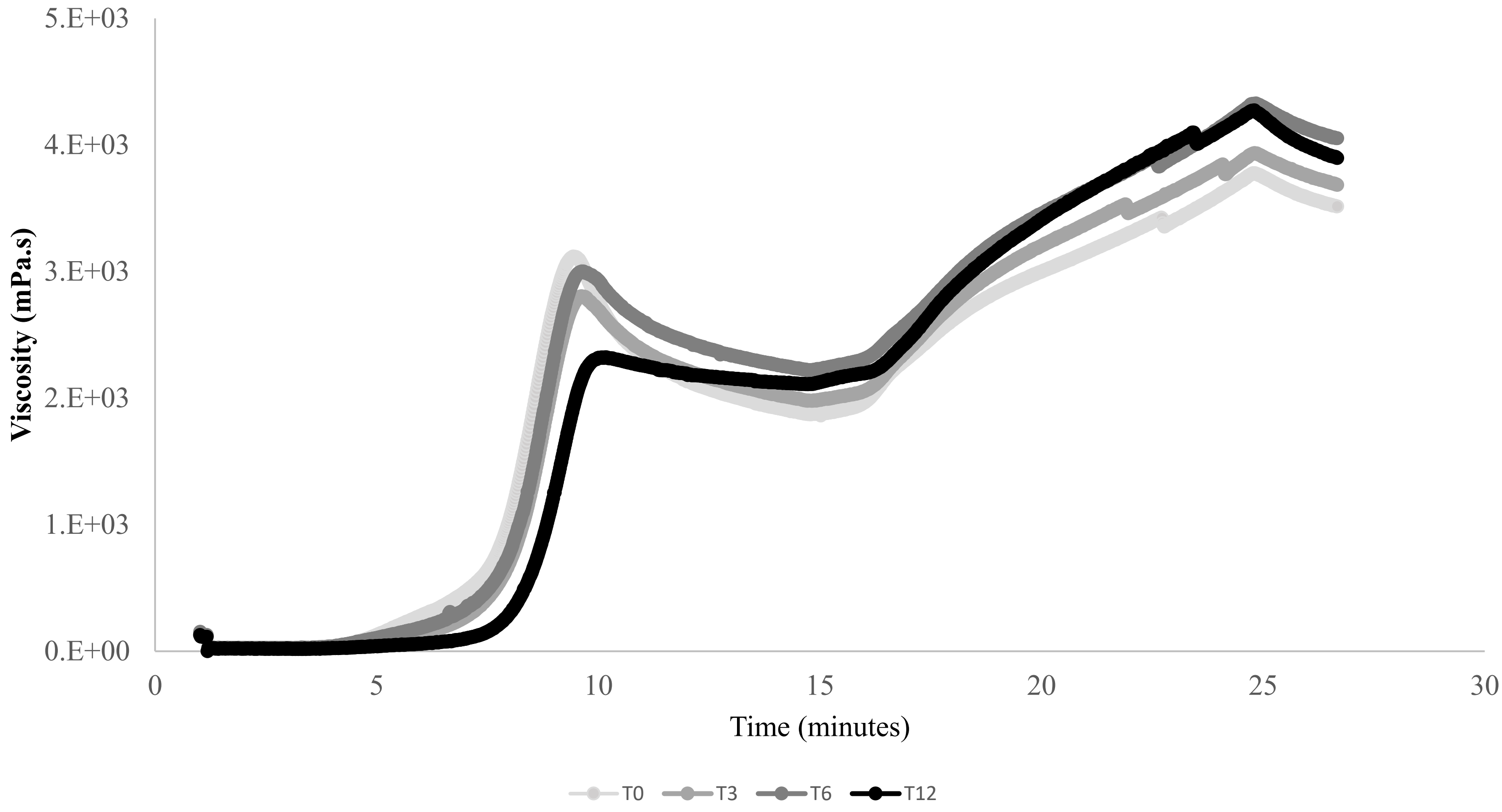

Figure S4. Rheological curves during storage in DRY conditions

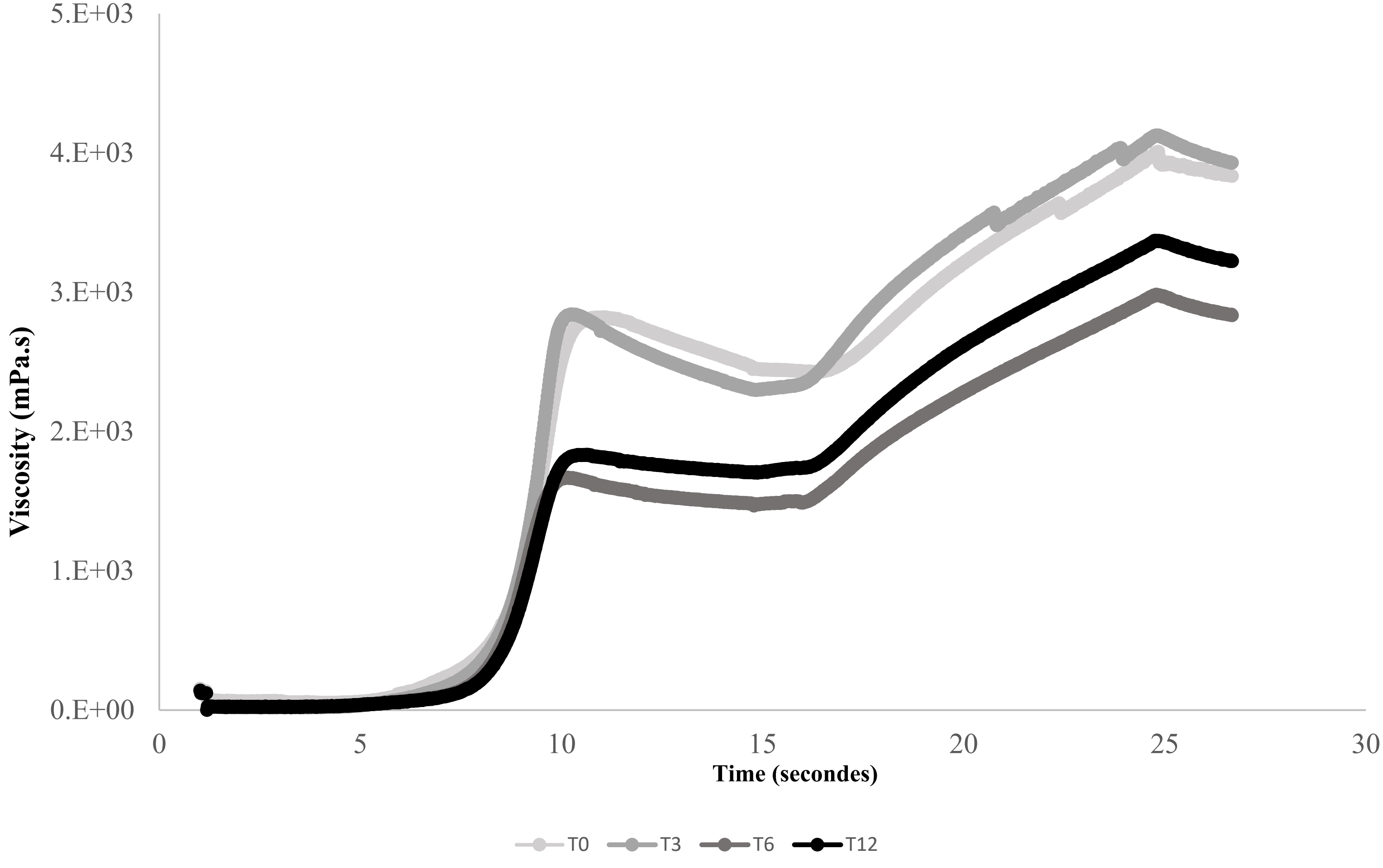

Figure S5. Rheological curves during storage in HSH conditions
